# A randomised controlled feasibility trial of family and social network intervention for young people who misuse alcohol and drugs: study protocol (Y-SBNT)

**DOI:** 10.1186/s40814-015-0004-4

**Published:** 2015-03-13

**Authors:** Judith Watson, Donna Back, Paul Toner, Charlie Lloyd, Ed Day, Louca-Mai Brady, Lorna Templeton, Sangeeta Ambegaokar, Steve Parrott, David Torgerson, Kim Cocks, Eilish Gilvarry, Paul McArdle, Alex Copello

**Affiliations:** 1 Department of Health Sciences, University of York, York, YO10 5DD UK; 2 School of Psychology, University of Birmingham, Edgbaston, Birmingham, B15 2TT UK; 3 Addictions Department, Institute of Psychiatry, 4 Windsor Walk, London, SE5 8AF UK; 4 Independent Research Consultant, London, UK; 5 Independent Research Consultant, Bristol, UK; 6 Birmingham Children’s Hospital, Steelhouse Lane, Birmingham, B4 6NH UK; 7 Northumberland, Tyne and Wear NHS Foundation Trust, Newcastle, UK; 8 Northgate Hospital, Morpeth, NE61 3BP UK; 9 Birmingham and Solihull Mental Health Foundation Trust, Birmingham, B1 3RB UK

**Keywords:** Feasibility study, Young people, Alcohol, Drugs, Acceptability, Social behaviour and network therapy, Randomised controlled trial, Patient and public involvement, PPI

## Abstract

**Background:**

A growing body of research has identified family interventions to be effective in treating young people’s substance use problems. However, despite this evidence, take-up of family-based approaches in the UK has been low. Key factors for this appear to include the resource-intensive nature of most family interventions which challenges implementation and delivery in many service settings and the cultural adaptation of approaches developed in the USA to a UK setting. This study aims to demonstrate the feasibility of recruiting young people to a specifically developed family- and wider social network-based intervention by testing an adapted version of adult social behaviour and network therapy (SBNT).

**Methods:**

A pragmatic, randomised controlled, open feasibility trial delivered in two services for young people in the UK. Potential participants are aged 12–18 years referred for drug or alcohol problems to either service. The main purpose of this study is to demonstrate the feasibility of recruiting young people to a specifically developed family and social network-based intervention. The feasibility and acceptability of this intervention will be measured by recruitment rates, treatment retention, follow-up rates and qualitative interviews. The feasibility of training staff from existing services to deliver this intervention will be explored. Using this opportunity to compare the effectiveness of the intervention against treatment as usual, Timeline Follow-Back interviews will document the proportion of days on which the main problem substance was used in the preceding 90-day period at each assessment point. The economic component will examine the feasibility of conducting a full incremental cost-effectiveness analysis of the two treatments. The study will also explore and develop models of patient and public involvement which support the involvement of young people in a study of this nature.

**Discussion:**

An earlier phase of work adapted social behaviour and network therapy (adult approach) to produce a purpose-designed youth version supported by a therapy manual and associated resources. This was achieved by consultation with young people with experience of services and professionals working in services for young people. This feasibility trial alongside ongoing consultations with young people will offer a meaningful understanding of processes of delivery and implementation.

**Trial registration:**

ISRCTN93446265; Date ISRCTN assigned 31/05/2013.

## Background

Early onset of drug use, including alcohol, in children and young people has been associated with later problematic use [1,2], as well as a range of other problems including risky sexual behaviour, injury, antisocial behaviour and changes in brain development [3-5]. Cannabis and alcohol are the two most commonly consumed drugs by young people in England, accounting for 90% of treatment admissions for young people [6]. Fifteen to 16 year olds in the UK have one of the highest rates of underage drinking and drunkenness in Western Europe, with the UK also amongst ten European countries with the highest proportion of students reporting smoking cannabis within the past 30 days [7].

Research has shown that substance use amongst young people can adversely affect relationships with parents, carers and other family members [8], but also that family involvement in interventions can influence the course of the problem in a positive way and act as a protection against substance-related problems [9]. As a consequence, a range of preventive and treatment approaches have focused on the family; and in the UK, there has been a strong focus on preventive programmes. A systematic review by Foxcroft and colleagues [10] identified the Strengthening Families Programme [11], developed in the USA, as the most promising, with positive outcomes in both the short and the long term. Emerging findings from the application of this model to the UK context have shown some promise [12].

The four most evaluated family interventions used with young people include ‘multisystemic therapy’, ‘integrated family and cognitive behavioural therapy’, ‘multidimensional family therapy’ and ‘brief strategic family therapy’ [13]. Reviews of evaluation studies have shown these approaches to be effective in reducing drinking and drug use amongst young people [13-15]. However, problems revolve around engagement of family (however defined), treatment decay and translating research into practice.

Firstly with regard to family engagement, services frequently have problems engaging individual family members [16]. Furthermore, the definition of ‘family’ is contested and carries implications for the delivery of family interventions [17]. Young people (YP) with substance use problems frequently come from disrupted and sometimes ‘complex’ families and may be looked after by single parents, grandparents, other relations or the state (e.g. Lloyd 1998; Boys et al. 2003 [2,18]). Traditional, systemic family approaches may be difficult to deliver in such situations. Secondly, researchers have pointed to the particularly rapid decay in treatment effect for adolescents’ drug and alcohol problems [13,15]. Thirdly, in terms of translating research into practice, the intensive training required [14] and the lengthy time required to deliver systemic family interventions can discourage practitioners from implementing them. UK National Treatment Agency statistics, for example, suggest that only 2% of interventions with the under-18 s consisted of ‘psychosocial and family work’ and 6% ‘psychosocial, family work and harm reduction’ [6].

The large majority of young people with substance misuse problems receive psychosocial interventions focused on the individual user that do not engage family members. Likewise, our recent survey conducted in the UK with services for adult family members showed that even those family interventions recommended by the National Institute for Health and Care Excellence (NICE) [19] such as Behavioural Couples Therapy [20] are rarely implemented [21].

A further potential barrier may be the need for cultural adaptation of approaches developed in the USA to a UK setting. There is growing awareness of the need to adapt evidence-based treatments to different cultural groups and healthcare settings in order to ensure successful implementation [22-24].

Social Behaviour and Network Therapy (SBNT) is an intervention developed in the UK shown to be effective with harmful drinkers [25], which utilises cognitive and behavioural strategies, helping clients build family and social networks which are supportive of behaviour change and goal attainment. The focus is on addressing substance misuse by engaging with a network of positive support for lifestyle change. SBNT may also have relevance to adolescent populations as it helps sustain engagement with vulnerable young people by widening the reach of the intervention beyond the traditional family to include supportive peers. The intervention aims to integrate strategies found to be effective in other family and network approaches, is based on the concept of enhancing social support for a positive change in substance use and consists of a series of core and elective topics used within sessions. Core strategies include motivational techniques, improving communication and coping mechanisms, and given the nature of substance misuse, developing a network-based relapse management plan. The therapeutic approach also has scope to address client-focussed elective areas, for example, educational requirements [26].

In an earlier phase of work, this research team adapted the current SBNT approach to produce a purpose-designed therapy manual and associated resources suitable for use with young people (Youth-SBNT or Y-SBNT). This was achieved by extensive and ongoing Patient and Public Involvement (PPI) with young people with experience of services, as well as consultation with treatment professionals working with young people. Whilst retaining the main aspects of the approach that were established to be relevant to the young population, the adaptation showed the need to pay particular attention to the identification of potential network support that is wider than the biological family and includes peers and important formal supports.

This study aims to demonstrate the feasibility of recruiting young people to receive this specifically developed family- and network-based intervention, establishing the acceptability of the intervention to both young people and their therapists.

## Methods/Design

### Design

**Figure 1:**
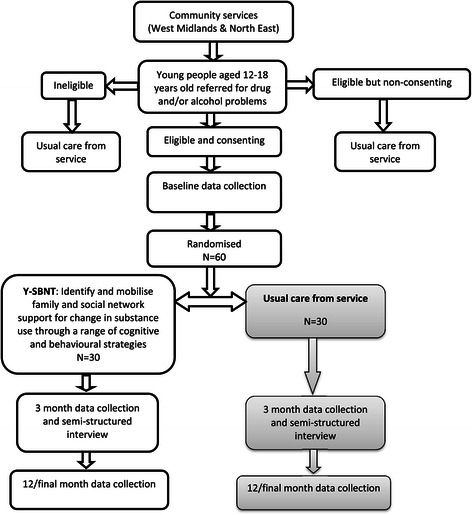
Flow diagram of Y-SBNT study.

The study is a pragmatic, randomised controlled, open feasibility trial in which a specifically developed family- and social network-based intervention is compared with treatment as usual for young people aged 12–18 years referred for treatment of drug or alcohol problems to two young people’s services. The study has been granted ethical approval by the National Research Ethics Committee West Midlands—Coventry & Warwickshire (Reference: 14/WM/0021). A full flow diagram for the study is shown in Figure 1.

### Aims of the study

- To demonstrate the feasibility of recruiting young people to a family- and social network-based intervention (Y-SBNT) across two service sites.
- To test the feasibility of training staff from existing young people’s addiction services to deliver the family and social network intervention.
- To evaluate the level of treatment retention amongst participants randomised to the family and social intervention compared to treatment as usual.
- To explore through qualitative interviews the participants’ views, acceptability and experiences of the intervention and the study process.
- To explore through qualitative interviews the views, acceptability and experiences of those attending treatment sessions as members of the young person’s network.
- To examine treatment effectiveness through 3- and 12-month outcome quantitative data.
- To explore cost effectiveness in preparation for a large definitive randomised controlled trial.
- To explore and develop models of patient and public involvement which support the involvement of young people in a study of this nature.

### Participants and setting

This feasibility study is being conducted in two UK centres that are representative of the types of young people services available across the UK:

- West Midlands, England: a service providing information, advice, treatment and support for problems related to the use of drugs, alcohol and other substances for people less than 18 years of age. The service consists of a multidisciplinary team offering individual and group services to young people with substance misuse problems and complex needs and delivers both assessment and treatment.
- North East of England: a specialist service for people less than 18 years of age that links with a number of generic youth services and with other primary care services such as general practitioners (GPs) and school nurses. Workers are mainly from the third sector who have extensive experience in addictions and youth development.

### Eligibility criteria

Young people (YP) are considered eligible if they:

- are aged 12–18 (males and females).
- have been newly referred and accepted for structured intervention for drug and/or alcohol problems by either treatment service during the period of recruitment.
- are willing and able to provide written informed consent and deemed Gillick competent (United Kingdom assessment made in regard to whether a child under 16 has the capacity to consent to treatment without parental or guardian consent) [27].

Young people are considered ineligible if they:

- have a concurrent severe mental illness that precludes them from active participation.
- have a severe physical illness that precludes them from active participation.

### Study procedures

#### Identification

All YP newly referred to the two treatment services during the recruitment period will be considered potential participants.

### Eligibility assessment

All referred YP will initially take part in an assessment session (routine part of the service referral and assessment processes) either at the treatment agency, home or usual place of treatment. Prior to conducting the assessment, a competency test based on the Gillick test is routinely administered to ensure that the young person is ‘competent’ to understand the implications of treatment as well as provide independent and valid consent. Those found to be appropriately referred and meeting the inclusion criteria will be deemed potentially eligible for the trial.

Eligible YP who do not wish to take part (i.e. unwilling to give consent) and those found to be ineligible will go on to receive usual care from the service. Where offered, reasons for non-participation will be collected to inform future studies.

Eligible YP and their parents/person with parental responsibility will be given a leaflet and patient information sheet (PIS) by the assessment staff. If they are interested after reading the materials, a meeting will be arranged with a researcher.

### Consent procedure

During the meeting with the researcher, those eligible and interested will have the study fully explained to them and be given the opportunity to ask questions. For those that agree to participate, written informed consent to participate in the trial will then be obtained.

The research process must ensure that informed decisions are made by YP and their parents/person with parental responsibility whether or not to take part in the trial. Competence is not related to age in a simple way but depends on a child’s ability to understand, weigh the options and reach an informed decision [28].

For the purpose of this study, the following will apply in line with the National Children’s Bureau (NCB) guidelines [29]:

- If consent is not forthcoming from a parent/person with parental responsibility but the young person (aged 12–15) does consent, they will still enter the trial.
- For those aged 16 and above, consent will be only be sought from the young person as those aged between 16 and 18 are presumed to be competent to give consent.
- If consent is given by a parent/person with parental responsibility but the young person does not consent, the young person will not enter the trial.

However, the possibly ‘chaotic’ and complex lives of many of these YP has to be considered and as such, discussion about consent in all cases will be handled in a sensitive manner. It is expected, on the basis of previous experience, that YP and their parents/person with parental responsibility decisions will usually be concordant. Details of up to three tracing contacts will be collected to assist with treatment retention and follow-up.

### Randomisation process

Participants will be randomised by a secure remote randomisation service run by the fully registered York Trials Unit, UK. Randomisation, stratified by centre, will allocate participants following baseline assessment to either treatment as usual (TAU—control treatment) or the adapted youth social network intervention (Y-SBNT).

### Intervention content

An initial phase of work was conducted in order to adapt the current social behaviour and network therapy approach [26] to produce a purpose-designed therapy manual. This was achieved through reviewing the current evidence-based literature and consultation with YP with experience of services and professionals working in YP services. This was conducted as part of PPI work through separate interviews, meetings and consultation events.

The resultant Y-SBNT will be delivered according to the therapy manual and consists of an initial appointment followed by five further approximately 50-min SBNT sessions over a maximum period of 12 weeks (aiming for one per week where possible or adapting frequency depending on clinical need). The intervention will be delivered by a therapist trained to do so and can be delivered at the treatment agency, the participant’s home or their usual place of treatment. A key strength of the SBNT approach is the primary focus on addressing drug and alcohol problems by engaging with a network of positive support for lifestyle change. Therefore, the first session will involve an introduction to the treatment method, identification of the young person’s social network and review of important people. Using this as a platform, subsequent core strategies of the adapted Y-SBNT approach will include identifying and developing achievable goals, developing network support and improving communication and coping mechanisms and developing a network-based relapse management plan. The participant is encouraged to invite members of their network to the treatment sessions. An important aspect is to ‘think network’ [26], and this involves the therapist always thinking about the young person within a social context and considering the impact of the YP substance-related behaviour upon others and the potential for the YP accessing and developing social support. The therapeutic approach has scope to address client-focussed elective areas, for example, educational requirements [26]. The Y-SBNT manual combines the most effective components of the SBNT intervention used in earlier studies with adults with substance use problems as well as those identified as important through consultation with YP, families and staff.

Those participants randomised to receive TAU will continue to receive their usual care delivered by their service, with appointments offered as required in the first 12 weeks. TAU generally focuses on engagement, description of substance use, current issues that the young person brings to sessions and which seem relevant to the substance use and practical matters such as housing or school exclusion. Participants allocated to TAU will be seen by a therapist not trained in Y-SBNT. Content of TAU sessions will be recorded and assessed at the end of the study.

Concurrent treatment will be available to both groups and can occur wherever there is a need for any identified medical treatment as and when necessary (e.g. treatment for attention deficit hyperactivity disorder).

### Training

Training in the intervention followed the format adopted in previous pilot work in this area [30]. Service therapists from the two participating clinical centres will be invited to express an interest in study participation and receive training in Y-SBNT. Given the pragmatic nature of this feasibility study, therapists will be selected on the basis of their expression of interest. At the time of writing, there are significant changes taking place in the services in the UK and staff turnover is high. Whilst random selection of therapists may be preferable, the risks associated with implementing such a process within routine services are deemed to be too high in terms of the maintenance of therapists’ engagement and retention within this early pilot phase.

Once selected, therapists will receive an initial 1-day training session to introduce the key concepts and procedures involved in the intervention. This will be supported by the original SBNT manual, specific written materials and resources to be used with young people. Staff will pilot the methods with a minimum of one clinical case prior to the commencement of the trial, until the intervention is being delivered with sufficient fidelity. Supervision thereafter will be provided fortnightly where each case is reviewed and discussed in detail with reference to the key components of the approach and available resources.

### Blinding

Due to the nature of the interventions used within this study, blinding of the participants, therapists and the researchers is not possible. However, baseline data will be collected prior to randomisation and those involved in the analysis of the data will be blinded to treatment allocation.

### Qualitative data

A key aim of this feasibility study is to establish the acceptability of the Y-SBNT intervention to the YP and their families and social network members. Participant interviews will be undertaken to explore these issues at 3 months post randomisation. The interviews will be semi-structured in nature and built on the work conducted as part of UKATT [31-34] and previous studies of SBNT with drug users (e.g. Copello et al. [30]) in order to explore perceptions of the effectiveness and utility of the new intervention. We aim to understand which elements of the treatment were beneficial and acceptable in the care of YP. This will complement the analysis of the quantitative data and identify ways in which Y-SBNT may need to be modified in preparation for a definitive trial.

Semi-structured interviews will also be conducted with members of the young person’s network who attend treatment sessions. All network members will be approached; and for those interested, interviews will be conducted by researchers either face-to-face or by telephone 3 months post randomisation. Interviewing network members will provide essential information about their thoughts on being involved in such a process including any impact it has on them and their relationship with the young person. This will also provide an opportunity to explore whether taking part in the treatment was acceptable to them and the perceived influence of their involvement on the young person’s substance use. A sample of ten individuals (one network member for every six YP participants at each service) will be interviewed.

In addition, the therapists delivering the Y-SBNT intervention and the TAU therapists will be interviewed at a single point; following the completion of all 3-month post-randomisation follow-up assessments. The interviews with Y-SBNT therapists will be used to explore a number of themes, including the training and implementation process, how the intervention differs from usual treatment and how easy it has been to engage YP and their social networks. These interviews will seek to identify potential problems with the delivery of the intervention and trial processes, with a view to ironing out any difficulties prior to a full trial. Interviews with service managers will cover similar ground, exploring issues of implementation but also broader questions about the popularity of the intervention amongst service staff.

All interviews will be conducted by trained researchers using the appropriate topic guide for the person being interviewed to promote consistency. The topic guides cover areas including satisfaction and acceptability of the intervention, aspects that were helpful or unhelpful from the participant’s perspective, the overall experience of the treatment and suggestions for improvement. Written informed consent will be gained prior to the qualitative interviews which will also be audio-recorded when permitted.

### Patient outcome measures

This study also provides an opportunity to explore effectiveness of the intervention when compared to TAU and inform any future trial of the most appropriate outcome measures. The main outcome will be substance use based on the Timeline Follow-Back (TLFB) interview [35] and in particular the proportion of days on which the main problem substance was used in the preceding 90-day period at each assessment point (3 and 12 months). The TLFB interview is based on a retrospective calendar review of each day’s consumption and has been validated and widely used with adolescent populations (e.g. Dennis et al. 2004; Waldron et al. 2007; Levy et al. 2004 [36-38]). Utilising the TLFB will allow the collection of detailed data on the full range of licit and illicit drugs used by participants. This will provide the opportunity to explore a range of substance use outcome measures, with a view to informing the outcome measure used in the full trial.

A number of secondary patient outcomes will also be measured. Behaviour, concentration, emotions and relationships with other people will be measured using the Strengths and Difficulties Questionnaire (SDQ) [39] which has been used extensively and demonstrates high levels of reliability and validity [40,41]. Given the emphasis on family and peer support of the intervention, structure and quality of social network support will be measured using the Important People Drug and Alcohol (IPDA) interview. This measure, with good internal consistency and validity, is considered useful to administer pre- and post-treatment to assess the degree to which social network changes have been achieved and to assess the extent to which these changes still need to be made [42]. Family environment will be measured using the 27-item relationship dimension of the Family Environment Scale (FES) [43] consisting of cohesion, expressiveness and conflict subscales (nine items each). It is designed to measure the atmosphere in the family household and will be used where appropriate to the circumstances of the participant. Extensive development and validation work has been conducted on the FES, with internal consistency, test re-test reliability and content and construct validity all reported as robust [44]. Health-related quality of life (HRQoL) will be assessed using the European Quality of Life—5 Dimensions—5 Levels (EQ-5D-5 L) instrument. EQ-5D is a standardised measure of health status developed by the EuroQol Group in order to provide a simple, generic measure of health for clinical and economic appraisal, where health is characterised on five dimensions (mobility, self-care, ability to undertake usual activities, pain, anxiety/depression) [45].

All these patient outcome measures will be collected during face-to-face meetings at baseline, 3 and 12 months post randomisation. For those participants randomised in the last 2 months of the recruitment period, final follow-up may take place within a 10- to 12-month period.

At the end of treatment sessions 1 and 3, both the young person and the therapist (Y-SBNT and TAU) will complete the 12-item Working Alliance Inventory (WAI) [46] in order to assess participant-therapist relationship.

The costs of delivering the two interventions will be calculated on the basis of resources used. Participant use of healthcare and social services as well as school attendance and engagement, self-reported crime and contact will be assessed through service-use questions at baseline and 12 months post randomisation. We will use utility values based on societal values using the York tariff [47]. Data will be also be collected at each treatment session on length of the event, who attended the session as a network member, the therapist involved, location and any materials used.

### Quality assurance of treatment delivery

Where consent is obtained, sessions in both treatment arms will audio-recorded and reviewed. This will ensure fidelity with the Y-SBNT manual and monitor the elements of TAU which will be carefully documented as part of the feasibility study.

### Recruitment

Recruitment is planned to last 6 months.

### Compliance and withdrawal

The issue of compliance has been explored with YP and also through an appraisal of academic reviews of family interventions with YP conducted as part of an earlier phase of work. Important strategies were identified to minimise drop out including factors related to the therapist style and orientation, structural factors, the actual therapy orientation and additional factors such as provision of a quick service response and use of mobile systems for appointment reminders and communication with YP. The intervention has, in addition, been developed to be flexible and delivered through outreach in a range of settings, e.g. schools, other services and participants’ homes.

Attrition from follow-up is a major threat to internal validity, and longitudinal studies of substance users frequently suffer from low follow-up rates; reflecting the ‘chaotic’ and complex nature of this group (e.g. Ziek et al. [48]). This study will draw on aspects of Scott’s Engagement, Verification, Maintenance and Confirmation (EVMC) model [49] to track and follow up participants in order to minimise such problems. These include building rapport with respondents, detailed locator information (including at least three tracing contacts), periodic reminders, use of the same researcher to carry out interviews at baseline, 3 months and 12 (final) months and high street shopping vouchers to compensate for their time completing interviews.

### Sample size calculation

Treatment service outcome data collected in the 6 months prior to the trial showed that one of the participating services currently receives approximately 45 new referrals per month and carries a caseload of over 200 clients. The second service had approximately 280 YP access in the last year. Drawing on National Treatment Agency statistics, it is expected that 90% of the sample will fall into the target age range [6].

As this is a feasibility study, the main purpose is to assess the acceptability and feasibility and to obtain information that would inform the design of a larger full-scale trial. Although a formal sample size calculation for a feasibility or pilot study is not required, we calculated the number of participants required so that an effect smaller than that desired in the main trial can be ruled out [50]. This will give a clear criterion on which to base the decision to go ahead with the main trial following the feasibility study.

For the main trial, we would want to detect 0.3 of a standard deviation between the two groups. This would require a sample size of approximately 350 patients. A pilot study of 32 patients would be sufficient to exclude this difference in the event of a zero or negative intervention effect. We would conclude, unless there was a clear explanation, that there was poor justification for moving towards a fully powered main trial as it would be unlikely that an effect size of 0.3 or greater would be found in a main trial. If there is a positive intervention effect in the pilot study, then we would conclude that the main trial is worthwhile providing adequate recruitment and follow-up rate was observed in the pilot study.

Given the patient population, a reasonably high level of attrition may be expected. We feel that we will need to recruit 60 participants to the trial to fully inform the design and sample size of the main study.

### Planned recruitment rate

It is expected that recruitment will be at the rate of ten patients per month (five from each site).

### Analysis

The main aims of this study are to demonstrate the feasibility of recruiting YP to a family- and social network-based intervention, test the practicalities of training staff to deliver it and, importantly, to evaluate the acceptability of the intervention to both the YP and the therapists.

### Qualitative analysis

Interviews will be digitally recorded (where consent is given) and fully transcribed. In line with our previous work involving qualitative evaluation of SBNT [51], analysis will be based on grounded theory methods [52]. Initial ideas will be identified and organised into higher order themes following group discussions and research group seminars. Some of the emerging findings will be presented to a selection of the original participants in order to check validity of the resulting interpretation.

### Primary statistical analysis

The feasibility of this current study and the potential for taking this forward to a large-scale study will be measured in conjunction with the qualitative data by examining recruitment rates, retention in treatment and follow-up completion rates.

The primary clinical outcome measure for the study will be the proportion of days on which the main problem substance was used in the preceding 90-day period at each assessment point. Analysis will be on an intention-to-treat basis using two-sided, 5% significance. The primary analyses will compare Y-SBNT with TAU, and participants will be analysed using a linear model accounting for clustering of participants within therapists. Missing data will be dealt with using sensitivity analyses to check the robustness of the primary analysis. As this is a feasibility study, the level of and reasons for missing data (where available) will be reported to inform any full-scale trial. An effect size will be calculated for average proportion of days used in the 90-day period, with an estimate of zero or above indicating that an effect size of 0.3 is plausible for the main trial.

### Secondary statistical analyses

Secondary outcomes will include SDQ and FES (relationship dimension) and EQ-5D-5 L, all measured at 3 and 12 months. Using the IPDA, we will calculate the same measure of social support calculated for the UK Alcohol Treatment Trial (UKATT) [53] in order to compare changes between baseline and follow-up for the young people. In addition, we will explore structural and functional components of the young persons’ social networks using a number of the IPDA sub-scales and changes again will be explored between baseline and follow-up. By means of the WAI, the relationship between the young person and therapist will be explored using descriptive statistics and correlation where appropriate.

### Economic analysis

The economic component of this trial will examine the feasibility of conducting a full incremental cost-effectiveness analysis of Y-SBNT compared to TAU. This will involve piloting a short questionnaire, analysing responses and calculating quality-adjusted life year (QALY) changes using EQ-5D-5 L. We would not expect to see significant changes between groups due to the small sample size in this pilot.

A simple questionnaire will measure participants’ use of healthcare retrospectively. The economic analysis will assess the feasibility of using such a questionnaire in the 12–18 population. The questionnaire will ask about primary care, hospital visits and hospital stays. In a full trial, resource use data will be multiplied by national average unit costs to calculate per participant costs in the 3-month period before the intervention and the 3-month period after receiving Y-SBNT or TAU.

Quality of life will be measured by EQ-5D-5 L at baseline and each follow-up time point. The use of EQ-5D-5 L enables the estimation of QALYs. Measuring health status using QALYs follows the recommendations of NICE [54] and enables the value for money afforded by treatment to be compared to a range of other healthcare interventions.

### Treatment fidelity analysis

Audio recordings of the treatment sessions will be used to rate the quantity and quality of the treatment components delivered by the therapists and their adherence to the Y-SBNT treatment manual. Using the extensive work conducted as part of the UKATT trial and the fidelity scale already developed [55], we will attempt to adapt this scale to measure fidelity to Y-SBNT. Frequency and quality of behaviour change techniques used and found to be integral to SBNT in previous work and including, for example, items such as a ‘social interpersonal focus in session’, ‘discussion of social support’ and ‘involving others’ will be rated. Depending on the sample available, this adapted scale will be tested on this population and we will attempt to test the validity and reliability as discussed by Tober et al. [55].

A number of TAU recordings will be assessed to try and identify the components of TAU. Recordings of 10%–20% sample of sessions across all therapists and both centres from the middle and end of the therapy will be assessed. Correlational analysis of the data derived from these ratings will be performed to detect protocol adherence and discriminability between the treatments.

### Public involvement

This study will allow us to explore ways in which young people with experience of using services can be involved in a study of this nature. As part of the significant PPI component of this study, the project team is actively involving a group of YP with a history of treatment for substance abuse throughout the research process. In phase 1, YP were supported to work alongside the research team in order to ensure that the intervention is acceptable and relevant to our target groups and reflects the views of service users and their families. During phases 2 and 3, YP will be involved in the project as it develops, including, for example, in the production of training materials, design of data collection tools, data analysis and interpretation, reporting and dissemination. Both YP and parents will also be supported to contribute to the Trial Steering Committee, and parents will also contribute to the development of the intervention through one or more consultation groups.

## Discussion

Adapting SBNT to a youth context in a manner that is acceptable to those receiving it and that can be readily and widely implemented in services for YP has great potential for impact. If the outcome of this study suggests that a large-scale multi-site trial is feasible, the opportunity to assess the clinical and cost effectiveness of this intervention could be highly valuable. In terms of benefits to society, should Y-SBNT prove clinically and cost effective and widely implemented, it would have a significant impact on the effectiveness of young people’s drug and alcohol treatment and associated health and social problems, thus reducing the costs to society. However, in addition to acceptability to YP, successfully implementing such an intervention is reliant on its acceptability to both those delivering it and those managing the services; an aspect this study team will be exploring. The need to design and trial a realistic intervention that can be readily delivered even in a climate of cuts for treatment services is a key aim of the present study.

Involving those who are the focus of the research can have a positive impact on what is researched, how research is conducted and the impact of research findings [56]. In recent years, there has been a theoretical and methodological shift amongst social researchers away from traditional approaches which saw children and young people mainly as objects of enquiry and towards a view that they are social actors, with their own unique views and insight into their own reality [57,58]. Although there is less of an evidence base in relation to children and young people’s involvement in research practice compared to adults [59], the case for their involvement has been explored in a number of publications [29,60-62].

An important feature of this study thus far has been the YP’s involvement in the development stage of the intervention which has allowed us to explore ways in which YP with experience of using drug and alcohol services can be involved in a study of this nature. This study is also piloting PPI with a cohort of young people whose voices are seldom heard in research. The project team had originally planned to recruit a group of young people who would be actively engaged throughout the project on an ongoing basis through a London-based ‘young advisors’ group. However, whilst we are continuing to pursue this option, we are also exploring more flexible and accessible options to enable more young people to be involved in the ways that work best for them. The valuable insights from this work will help to shape future PPI should a larger trial take place. Working collaboratively with young people involved in this study, we will seek to develop a model of PPI which addresses some of the issues emerging from this pilot. Designing a model for involvement with young people will enable us to reflect together on emerging learning, ensure that PPI in a larger trial is embedded from the outset and also contribute to the emerging evidence base and current debates around PPI with children and young people [63].

In addition to the development of a model of PPI, the exploration of the feasibility aspects of the trial, the adaptation of the approach and the ability to recommend moving to a definitive trial or not, there are a number of other resulting learning opportunities linked to research measurement. The measurement of substance use in this young client group is not without challenge. The complex nature of poly-substance use and the use of legal as well as illegal substances in complex patterns can create challenges when attempting to obtain accurate and clinically meaningful measurement and monitoring change and improvement in this group. The best ways to assess these will be explored with the use of the TLFB method which allows a detailed exploration of all substances used over the 3-month time period. In addition, the appropriateness of certain family-focused measures for this client group will also be explored. Some of the living circumstances of young people with substance misuse problems are away from family of origin or indeed away from family life (e.g. living in hostel accommodation at a very early age). How measures based on more traditional concepts of family life are perceived by the participants and how relevant the questions are to them is an important area to explore and understand further.

Finally, the mixed methods used will allow for a meaningful understanding of processes of delivery and implementation that to date tend to be neglected in clinical research.

## Trial status

At the time of submission, participants are actively being recruited into this study. First participant was randomised 30/05/2014.
